# Comprehensive Volatilome Signature of Various Brassicaceae Species

**DOI:** 10.3390/plants12010177

**Published:** 2023-01-01

**Authors:** Igor Lukić, Nina Išić, Dean Ban, Branka Salopek Sondi, Smiljana Goreta Ban

**Affiliations:** 1 Institute of Agriculture and Tourism, Karla Huguesa 8, 52440 Poreč, Croatia; 2 Centre of Excellence for Biodiversity and Molecular Plant Breeding, Svetošimunska 25, 10000 Zagreb, Croatia; 3 Department of Molecular Biology, Ruđer Bošković Institute, Bijenička Cesta 54, 10000 Zagreb, Croatia

**Keywords:** *Brassica*, lipoxygenase pathway, green leaf volatiles, glucosinolate hydrolysis, isothiocyanates, volatilome

## Abstract

To investigate in detail the volatilomes of various Brassicaceae species, landraces, and accessions, and to extract specific volatile markers, volatile aroma compounds were isolated from plant samples by headspace solid-phase microextraction and analyzed by gas chromatography/mass spectrometry (HS-SPME-GC/MS). The data obtained were subjected to uni- and multivariate statistical analysis. In general, two cabbage (*Brassica oleracea* L. var. *capitata*) landraces emitted the lowest amounts of volatiles generated in the lipoxygenase (LOX) pathway. Wild species *Brassica incana* Ten. and *Brassica mollis* Vis. were characterized by relatively high *trans*-2-hexenal/*cis*-3-hexen-1-ol ratio in relation to other investigated samples. A Savoy cabbage (*Brassica oleracea* L. var. *sabauda*) cultivar and three kale (*Brassica oleracea* L. var. *acephala*) accessions exhibited particular similarities in the composition of LOX volatiles, while the LOX volatilome fraction of *B. incana* and *B. mollis* partially coincided with that of another wild species, *Diplotaxis tenuifolia* L. Regarding volatiles formed in the glucosinolate (GSL) pathway, Savoy cabbage and wild species *B. incana*, *B. mollis*, and *D. tenuifolia* showed more intense emission of isothiocyanates than cabbage and kale. *Diplotaxis tenuifolia* showed a rather limited production of nitriles. The results of this study contribute to the general knowledge about volatile composition from various Brassicaceae species, which could be exploited for their better valorization. Future studies should focus on the influence of various environmental, cultivation, and post-harvest factors to obtain data with a higher level of applicability in practice.

## 1. Introduction

The Brassicaceae family includes 338 genera and 3709 species [1], among which the genus *Brassica* is one of the most important, since it includes many species domesticated by humans that have agricultural and economic relevance [2]. Today, Brassicaceae species are appreciated by consumers and are among the most commonly consumed vegetables globally due to their high nutritional value and abundance in various valuable bioactive compounds and elements, including carotenoids, tocopherols, ascorbic acid, glucosinolates, phenols, and others [3,4,5].

Among specialized plant metabolites in Brassicaceae plants, volatile organic compounds have many important biological properties and functions. They can act as signaling molecules within and between plants, provide protection by deterring herbivores or attracting their predators, and contribute to plant resistance to damage and stress [6,7,8,9,10,11,12,13]. Several biosynthetic pathways are responsible for their formation, the most important being the lipoxygenase (LOX), glucosinolate (GSL), and methylerythritol phosphate (MEP) pathways in which the so-called green leaf C_6_ and C_5_ volatiles, isothiocyanates (ITCs), and terpenoids are formed, respectively [10]. The entire profile of volatile compounds emitted by a plant that can be detected is also called the volatilome.

Volatile compounds from Brassicaceae species show important biological functions. For example, it was shown that main volatiles generated in the LOX pathway, *cis*-3-hexenol and its related aldehydes, have fungicidal and bactericidal activities [14], while volatile ITCs show antioxidant and cancer-preventing effects [15,16,17]. Besides having physiological functions in plants and beneficial effects on human health, volatile compounds are also important because they determine the specific flavor and aroma of Brassicaceae vegetables [18].

Despite several valuable reports being published to date, volatile compounds of Brassicaceae species have been investigated to a much lower extent than those of some other agricultural and food products, such as grapes and wine, olive oil, tomato, and others. Most of these reports focused on the world-known species, such as cabbage (*B. oleracea* L. var. *capitata*) [10,19,20,21] or rocket salad (*D. tenuifolia* L. (DC) and *Eruca sativa* L.) [16,17,22,23,24], while volatile profiles of species such as Savoy cabbage (*B. oleracea* var. *sabauda*) or kale (*B. oleracea* var. *acephala*) and especially rare wild relatives, such as *B. incana* Ten. and *B. mollis* Vis., have been investigated rarely or not at all. Most of the studies on volatiles in Brassicaceae plants referred only to a single or a small number of species, varieties, or accessions, without a comparative differentiation of larger numbers of groups. Many studies used commercially available samples from markets without information about their origin, meaning the determined variations possibly did not originate only from genotype, but also from variable environmental and growing conditions, transport, storage, and others. Finally, in most studies, a relatively low number of volatile compounds were identified, which was also a limiting factor in obtaining comprehensive information about their composition in Brassicaceae plants.

The aim of this study was to investigate in detail the volatilomes of various Brassicaceae species and to extract species-specific volatile markers. The intention was to compare the volatilomes of interesting, but rare and less or non-studied *Brassica* varieties (*B. oleracea* var. *sabauda* and *B. oleracea* var. *acephala*) and *Brassica* wild relatives (*B. incana* Ten. and *B. mollis* Vis.) to those of globally widespread Brassicaceae species, such as cabbage and wall rocket. The additional aim was to investigate the variability within a particular species by comparing various landraces and accessions. The obtained results could contribute to a better understanding of the potential of certain Brassicaceae species, varieties, and accessions in responding to stimuli from the environment, as well as understanding their typical sensory properties.

## 2. Results and Discussion

A HS-SPME-GC/MS method used in this study allowed for the identification of 95 volatile compounds from various biochemical pathways. Brassicaceae and other plants emit negligible amounts of volatiles in their natural state, so HS-SPME was carried out after disruption of plant tissue simulating wounding stress, which allowed naturally present enzymes to come into contact with substrates and produce volatiles [10,25]. Extraction time was relatively short to avoid possible subsequent reactions between volatile compounds, as well as their loss during eventual sample pretreatment, as proposed by Liu et al. [10]. Significant differences between the investigated species, landraces, and accessions were found for the majority of the investigated volatiles (Table 1).

### 2.1. Volatile Compounds from the Mevalonic Acid (MVA) and Methylerythritol Phosphate (MEP) Pathways

Volatile terpenoids have important roles in plant defense against herbivores and pathogens. They attract arthropods that prey on or parasitize herbivores, have a role in reproduction by attracting pollinators and seed disseminators, and play a part in responding to various stresses [26,27]. Terpenoids originate from two main precursors, isopentenyl diphosphate (IPP) and dimethylallyl diphosphate (DMAPP), from two alternate biosynthetic pathways: the cytosolic mevalonic acid (MVA) pathway, in which mainly sesquiterpenes are produced, and the plastidial methylerythritol phosphate (MEP) pathway, which is mainly responsible for the production of monoterpenes [28]. Many enzymes are involved in the mentioned biochemical reactions. For example, the formation of IPP and DMAPP from pyruvate and D-glyceraldehyde 3-phosphate in the MEP pathway consists of seven main enzymatic steps [29].

In this study five terpenoids were identified, including only a single monoterpene, eucalyptol, and a single sesquiterpenoid, perhydrofarnesyl acetone (Table 1). The other three compounds were norisoprenoids, formed in a parallel biosynthetic branch mainly by cleavage of carotenoid precursors [30]. Previous studies reported higher numbers of terpenes identified in cabbage [10,19], kale [31], salad rocket (*E. sativa* L.) [23], and other *Brassica* species not included in this study [10]. The highest amount of β-ionone, a C_13_-norisoprenoid with an odor reminiscent of violets, was found in *B. mollis*. Two cabbage landraces contained the lowest amounts of both β-ionone derivatives, but were, together with Savoy cabbage, most abundant in isophorone, known for its characteristic peppermint-like odor. Kale accession KAL3 and the investigated wall rocket accession emitted the highest levels of perhydrofarnesyl acetone, a volatile with a floral scent.

### 2.2. Volatile Compounds from the Lipoxygenase (LOX) Pathway

Volatile compounds formed in the lipoxygenase (LOX) pathway are also known as ‘green leaf volatiles’ since they provide vegetables with characteristic odor notes reminiscent of green leaves and grass. They are emitted in high amounts from plants under stressed conditions, such as wounding or pathogen infection, play a role in plant defense, and serve for communication with insects and other neighbor plants [25,32].

LOX volatile compounds mainly belong to the chemical classes of aldehydes, ketones, alcohols, and esters. Following the activation of the LOX pathway during plant development or by a stressor, sequential action of several enzymes is initiated. Lipoxygenases (LOX) catalyze regiospecific dioxygenation of long-chain linoleic (C_18:2_) and linolenic (C_18:3_) fatty acids to form corresponding hydroperoxides, which then serve as substrates to hydroperoxide lyases (HPL) to produce saturated and unsaturated volatile C_6_ or C_9_ aldehydes. After that, aldehydes may be converted to other isomers by isomerases, while alcohol dehydrogenases (ADH) and alcohol acyl transferases (AAT) partially transform them to alcohols and esters, respectively [25].

In plants, C_6_ dominates over C_9_ forms. In another branch of the pathway, hydroperoxides are transformed to C_5_ analogues. Several isoforms of LOX occur in most plants, which is most probably the case for other enzymes as well. For example, six LOX isoforms were detected in Arabidopsis [33].

In this study, the most abundant LOX compounds in all the investigated Brassicaceae species were C_6_ volatiles (Table 1). *Cis*-3-hexen-1-ol was found in the highest amount among alcohols in all the samples, followed by 1-hexanol and *trans*-3-hexen-1-ol. High levels of C_6_ aldehydes were also noted, primarily *trans*-2-hexenal, followed by hexanal. Much lower, but still relatively high amounts of other hexenals were also detected. The most abundant among C_5_ compounds were alcohols *cis*-2-penten-1-ol and 1-penten-3-ol. Considering that two of the most abundant LOX generated volatiles were *trans*-2-hexenal and *cis*-3-hexen-1-ol, the significant activity of LOXs, isomerases, and ADHs was obvious, while the absence of esters suggested that the loads of AATs were not significant, or they were not particularly active. Previous studies reported similar relations among the major LOX volatile compounds in cabbage [10], kale [31], salad rocket (*E. sativa* L.) [23], and other *Brassica* species [10], although with particular dissimilarities.

In this study, the investigated cabbage landraces were generally the least abundant in LOX volatile compounds and this feature differentiated them well from most of the other samples (Table 1). This was in accordance with the results from Liu et al. [10], who also found lower levels of LOX volatiles in cabbage in relation to particular other *Brassica* species. Compounds found in cabbage in the highest amount compared to other samples were 2-ethyl-1-hexanol in both landraces and *cis*-3-hexenal in landrace Brgujski. Among the LOX compounds, two cabbage landraces differed among each other only with respect to *cis*-3-hexenal and hexanal, with higher levels of the former found in Brgujski and that of the latter in Žminjski landrace.

*Brassica incana* and *B. mollis* differed from kale accessions and Savoy cabbage mostly by higher amounts of *trans*-2-butenal and lower amounts of *trans*-3-penten-2-one, 3-ethyl-1,5-octadiene, a non-identified alkene (*m*/*z* 55, 70, 41), 3-pentanone, and 1-hexanol. The highest amount of *trans*-2-hexenal among all the investigated samples was found in *B. incana*, while *trans*,*trans*-2,4-heptadienal reached the highest level in *B. mollis*. These two wild species differed between each other with respect to *cis*-3-hexen-1-ol, 1-penten-3-ol, *trans*-2-penten-1-ol, 2-methyl-2-pentenal, *cis*-4-heptenal, the non-identified alkene (*m*/*z* 55, 70, 41), *trans*-2-hexenal, *trans*-2-pentenal, *cis*-2-hexenal, and *trans*,*trans*-2,4-hexadienal that were found in higher amounts in *B. incana*, while *trans*,*trans*-2,4-heptadienal and *trans*-6-nonenal were more abundant in *B. mollis*.

Savoy cabbage stood out with the highest amounts of 3,5,5-trimethyl-3-cyclohexanone, *trans*-3-penten-2-one, 3,4-nonadiene, and especially *trans*-3-hexen-1-ol. These were the only volatile compounds that differentiated this species from all the three kale accessions.

Kale accessions showed a particular degree of mutual differentiation based on LOX volatile compounds, with KAL1 containing the highest *trans*-2-octenal and the lowest 6-methyl-5-hepten-2-ol and 2-methyl-2-pentenal amounts, KAL2 emitting the highest levels of *trans*-2-butenal, *trans*,*cis*-2,4-heptadienal, hexanoic acid and nonanoic acid, and the lowest levels of 1-hexanol and octanal, and KAL3 being characterized by the highest amounts of *cis*-3-hexen-1-ol, 6-methyl-5-hepten-2-ol, 3-pentanone, 1-hexanol, *trans*-2-hexen-1-ol, and *cis*-3-hexenyl isovalerate. Many of these compounds were found in the highest amount in kale accessions among all the investigated samples. The three investigated kale accessions were differentiated well by the levels of 6-methyl-5-hepten-2-ol and 1-hexanol, while other LOX compounds differentiated particular kale accession pairs well. Together with Savoy cabbage, kale accessions were more abundant in total LOX volatile compounds compared to the wild species and cabbages, which roughly implied higher LOX enzymatic activity in these samples.

Wall rocket accession was characterized by higher amounts of methyl-substituted unsaturated volatile compounds generated in the LOX pathway, such as 3,5,5-trimethyl-2-hexene, 6-methyl-5-hepten-2-ol, 2-methyl-2-pentenal, 5-methyl-3-heptene, and 2,2-dimethyl-3-hexene, but also of *trans*-3-hexenoic acid, octanal, *trans*,*trans*-2,4-hexadienal, and *trans*,*trans*-2,4-nonadienal.

The average ratio of the amounts of the two major LOX volatiles *trans*-2-hexenal/*cis*-3-hexen-1-ol was by far the highest in *B. mollis*, 3.2, followed by *B. incana* with 1.7. Since both volatiles are derived from the same precursor *cis*-3-hexenal [25], this implied that the relative activity of isomerases in *B. mollis* and *B. incana* overpowered that of ADHs. The opposite was probable in kale accessions and Savoy cabbage, for which lower average *trans*-2-hexenal/*cis*-3-hexen-1-ol ratios of 0.5, 0.3, 0.6, and 0.4 were determined, respectively. Medium ratios of 0.9, 1.3, and 1.2 were determined for cabbage landraces Brgujski and Žminjski and for the wall rocket accession, respectively. The accumulation of *cis*-3-hexenal in cabbage landrace Brgujski was possibly an indicator of a slowdown of the LOX cascade at that point. High amounts of hexanal in cabbage Žminjski and in particular other samples, pointed to the possibility of higher availability of linoleic acid, the main substrate in this branch of the LOX pathway [25]. Obviously, many differences and similarities in LOX volatile composition were observed between various groups of the investigated samples, while each of them was characterized by certain particularities. Such a range of features certainly resulted from a complex interplay between various enzymes and their isoforms, additionally conditioned by the availability of substrates, which was determined by genotype and was evidently unique for each variety or accession. It was previously reported that various *Brassica* species significantly differ in the number and type of genes that regulate the fatty acid metabolism, i.e., the LOX pathway [10].

**Figure 1:**
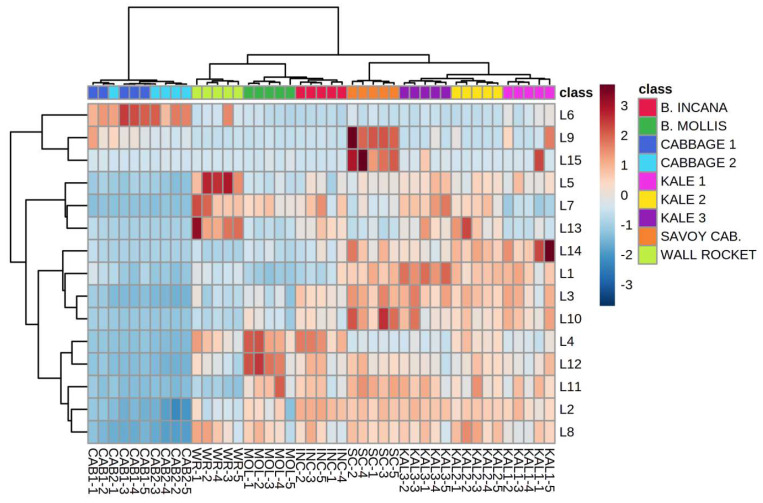
Clustering of Brassicaceae species by hierarchical cluster analysis (HCA) based on the composition of volatile compounds generated in the lipoxygenase (LOX) pathway. The rows in the heatmap diagram represent compounds and the columns represent samples. Codes indicating compounds correspond to those reported in Table 1. Colors of the heatmap cells indicate a low (dark blue), medium (white), and high (dark red) abundance of a particular compound.

Hierarchical clustering analysis (HCA) was conducted on a reduced dataset which included all the samples grouped into nine groups (species, varieties, accessions) and LOX generated volatile compounds as variables. Clear clustering of the groups was obtained after including the first 15 selected variables, which is shown on a heatmap diagram in Figure 1. Particular species, varieties, and accessions were related mostly to the characteristic volatile compounds previously determined by ANOVA (Table 1). Cabbage landraces were characterized by abundance in 2-ethyl-1-hexanol (L6) but were the poorest in other LOX volatiles. They formed a separate cluster and differed most from the other species based on the LOX fraction of the volatilome. Wall rocket samples were clustered together mostly based on the highest amounts of 3,5,5-trimethyl-2-hexene (L5), 6-methyl-5-hepten-2-ol (L7), and 2-methyl-2-pentenal (L13). *Brassica incana* and *B. mollis* were clearly richer in *trans*-2-butenal (L4), while the latter also emitted the highest levels of *trans*,*trans*-2,4-heptadienal (L12). These two wild species were clustered together and were the closest and most similar to the wall rocket accession. Kale accessions, especially KAL3, were clustered mainly based on high *cis*-3-hexen-1-ol (L1) amount. Additionally, they were grouped rather close, with KAL2 and KAL3 being more similar to each other. Savoy cabbage contained mostly 3,5,5-trimethyl-3-cyclohexanone (L9) and *trans*-3-hexen-1-ol (L15). Judging from their relatively close grouping, the overall composition of LOX volatiles in kale accessions turned out to be more similar to that of Savoy cabbage than to those emitted by the other investigated species.

**Figure 2:**
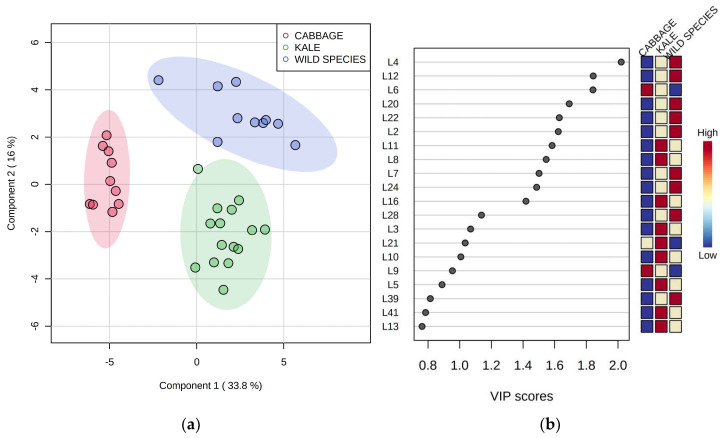
(**a**) Differentiation of Brassicaceae species in two-dimensional space by partial least squares–discriminant analysis (PLS–DA); (**b**) variable importance in projection (VIP) scores of volatile compounds (variables) generated in the lipoxygenase pathway most useful for the differentiation. Codes indicating compounds correspond to those reported in Table 1.

To better identify volatile compounds characteristic for particular species, which are therefore most useful for their mutual differentiation, the data on volatiles were subjected to partial least squares–discriminant analysis (PLS-DA). Savoy cabbage and wall rocket were excluded from PLS-DA since they were represented by a single variety or accession, respectively, while two cabbage landraces, three kale accessions, and two wild species *B. incana* and *B. mollis* were grouped into three corresponding groups, respectively. The PLS-DA differentiation according to species based on LOX volatiles data was very successful, as can be seen in Figure 2a. Twenty LOX volatile compounds with the highest variable importance in projection (VIP) scores are listed in Figure 2b. A compound with the highest VIP score (>2.0) was *trans*-2-butenal (L4), which was most abundant in two wild species and least abundant in cabbage. Several other LOX volatile compounds with relatively high VIP scores (>1.4) were confirmed to be most characteristic for wild species, such as *trans*,*trans*-2,4-heptadienal (L12), *trans*-2-hexenal (L20), *trans*-2-pentenal (L22), 1-penten-3-ol (L2), 6-methyl-5-hepten-2-ol (L7), and 1-penten-3-one (L24). High amounts of 3,4-nonadiene (L11), *trans*,*cis*-2,4-heptadienal (L8), and *cis*-4-heptenal (L16) served for the differentiation of kale, while the main marker for cabbage among LOX compounds was 2-ethyl-1-hexanol (L6).

### 2.3. Volatile Compounds from the Glucosinolate (GSL) Pathway

Brassicaceae vegetables contain glucosinolates (GSLs), a group of sulfur-containing compounds of variable structure that have important roles in plants’ defense against various stressors. After the disruption of plant cells, GSLs come into contact with endogenous myrosinases, β-D-thioglucosidases, whose activity results in the release of various hydrolysis products, such as volatile ITCs, nitriles, and epithionitriles [20,34]. Isothiocyanates are considered the most important volatile compounds deriving from the GSL pathway since, besides their key contribution to the sharp odor, bitterness, and pungency of various Brassicaceae vegetables [35], they exhibit significant beneficial effects on human health [15]. Brassicaceae vegetables can also contain epithiospecifier proteins that affect the hydrolysis of GSLs in a way that may result in releasing more nitriles or epithionitriles than ITCs [20,36].

Among volatile compounds generated in the GSL pathway, cabbage landrace Brgujski emitted the highest amounts of particular nitriles, such as 4-methylpentanenitrile and 3-(methylthio)butylnitrile, while it also had a high amount of hexanenitrile together with the other cabbage landrace Žminjski (Table 1). Only 4-methylpentanenitrile and 3-(methylthio)butylnitrile were found to mutually differentiate the two cabbages, both being more abundant in Brgujski landrace. Both cabbages were characterized by a relatively low total ITCs/nitriles ratio compared to the investigated wild species, although not significantly different from that of *B. incana*. As the total peak area of nitriles was not higher than that found in the majority of other samples, the low ratios found could be attributed to relatively low production of ITCs, as a result of weak myrosinase activity.

All three kale accessions released relatively high amounts of methyl thiocyanate, while among them, KAL1 stood out with higher amounts of 4-methylpentanenitrile, carbon disulfide, and benzyl nitrile. For several compounds, differences were also observed between different pairs of kale accessions. Similar to cabbages, kale accessions were characterized by relatively low ITCs/nitriles ratios.

Several ITCs were found in the highest amount in Savoy cabbage, namely allyl isothiocyanate, previously reported as a major isothiocyanate in various *Brassica* species like cabbage [19,21] and kale [31], as well as an unidentified isothiocyanate derivative (RI 1449, *m*/*z* 99, 41, 39, 72) and 2-methylbutyl isothiocyanate, with the highest amount of phenethyl isothiocyanate shared with *B. mollis*.

The GSL volatiles found in higher amounts in *Brassica* wild species *B. incana* and *B. mollis* included 4-mercaptophenol, 2,4-pentadienenitrile, benzenepropanenitrile, 3-butenyl isothiocyanate, 2-ethylthiophene, 4-(methylthio)butylnitrile, and benzyl nitrile, although without statistical significance in relation to some particular samples in a few cases. The two species mutually differed based on the amounts of several GSL compounds, such as, for example, benzenepropanenitrile among nitriles and 3-butenyl isothiocyanate among ITCs, which were both found in the highest amount in *B. mollis* among all the investigated samples. Several other GSL-derived compounds were found in higher amounts in *B. mollis* than in *B. incana*, while *B. incana* was the most abundant in 2-ethylthiophene among all the investigated samples. *B. mollis* was characterized by the highest sum of both ITCs and nitriles among all the samples, mostly due to the high proportions of benzenepropanenitrile and 3-butenyl isothiocyanate, respectively, which implied that the enzymatic activity in this species was relatively high. It is worth mentioning that relatively high ITCs/nitriles ratios observed in *B. incana* and especially *B. mollis* when compared to the cabbages and kales were mainly due to higher total ITCs amounts, possibly due to stronger myronase activity. *Brassica incana* and *B. mollis* emitted rather low amounts of methyl thiocyanate, carbon disulfide, 2-methylbutyl isothiocyanate, 3-(methylthio)butylnitrile, allyl nitrile, an unidentified isothiocyanate derivative (RI 1837, *m*/*z* 99, 71, 72, 59), and dimethyl disulfide, which differentiated them well from cabbage landraces, kale accessions, and a Savoy cabbage cultivar, while they shared most of the mentioned features with wall rocket.

Wall rocket exhibited several characteristic differences with respect to the other investigated vegetables. It emitted the highest levels of 4-methylpentyl isothiocyanate, carbon disulfide, and benzyl isothiocyanate, as well as an unidentified cyclic sulfur compound (IR 1846, *m*/*z* 88, 116, 117, 60), iberverin, and erucin, which were found in traces or not at all in other samples. Benzyl isothiocyanate and erucin accounted for most of the sum of ITCs in this species. Erucin (4-(methylthio)butyl isothiocyanate), which derives from glucoerucin, one of the major GSLs found in rocket leaves, was previously reported as the main volatile ITC in rocket salad (*E. sativa* and *D. tenuifolia*) [16,17,22,23]. The investigated wall rocket accession had the highest ratio of total ITCs/nitriles. Although it contained a total amount of ITCs that was higher than those found in cabbage landraces and kale accessions, a high ratio was mostly due to the very low total amount of nitriles (Table 1). This implied a weaker influence of epithiospecifier proteins that divert the GSL pathway towards producing more nitriles or epithionitriles at the expense of ITCs [20,36]. Several compounds, such as 2-methylbutyl isothiocyanate and an unidentified isothiocyanate derivative (RI 1837, *m*/*z* 99, 71, 72, 59), were not detected in the investigated wall rocket accession.

**Figure 3:**
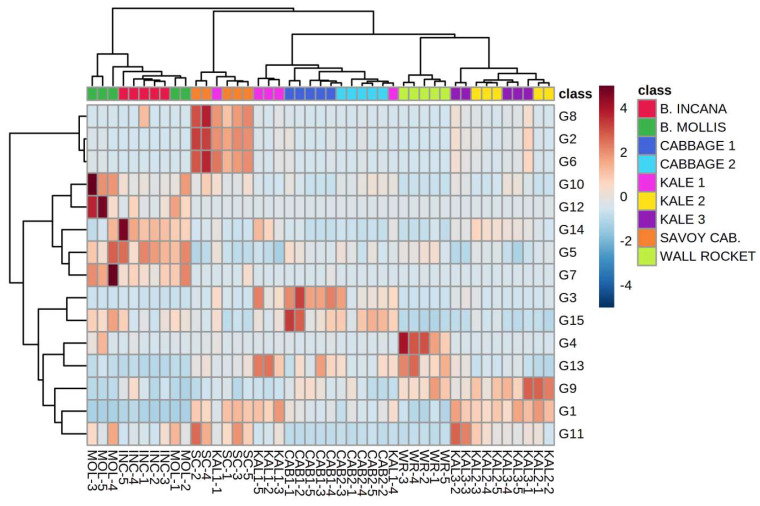
Clustering of Brassicaceae species by hierarchical cluster analysis (HCA) based on the composition of volatile compounds generated in the glucosinolate (GSL) pathway. The rows in the heatmap diagram represent compounds and the columns represent samples. Codes indicating compounds that correspond to those reported in Table 1. Colors of the heatmap cells indicate low (dark blue), medium (white), and high (dark red) abundance of a particular compound.

HCA was conducted on a reduced dataset which included all the samples grouped into nine groups (species, landraces, and accessions) and GSL generated volatile compounds as variables. Relatively good clustering was obtained including the first 15 selected variables, although particular landraces and accessions within particular species were not completely distinguished (Figure 3). KAL1 showed significant deviations since it was not clustered together with other kale accessions and its samples were scattered rather randomly among those of different species. Wild species *B. incana* and *B. mollis* were clustered together, mostly by high amounts of 4-mercaptophenol (G5) and 2-ethylthiophene (G14) that were more abundant in the former, and benzenepropanenitrile (G10), 3-butenyl isothiocyanate (G12), and 2,4-pentadienenitrile (G7) that were more abundant in the latter, respectively. Savoy cabbage was clearly characterized by higher amounts of an unidentified isothiocyanate derivative (RI 1449, *m*/*z* 99, 41, 39, 72) (G8), 4-methylthiazole (G2), and allyl isothiocyanate (G6), same as cabbages by the amounts of 4-methylpentanenitrile (G3) and hexanenitrile (G15). 4-Methylpentyl isothiocyanate (G4) and carbon disulfide (G13) were most characteristic for wild rocket, while methyl thiocyanate (G1) and 1-allyl-1-cyclohexanol (G9) were most responsible for clustering KAL2 and KAL3 samples together. Interestingly, and contrary to the case of LOX volatiles, wall rocket turned out to be most similar to the mentioned two kale accessions. Together with the cabbages, they formed a larger cluster distant from Savoy cabbage, and especially from wild species *B. incana* and *B. mollis*. It should be noted that particular volatiles, such as erucin (G16) and iberverin (G19) that were almost exclusively found in wild rocket, were excluded by the HCA model, since they were not useful for differentiation of the other investigated samples among each other.

**Figure 4:**
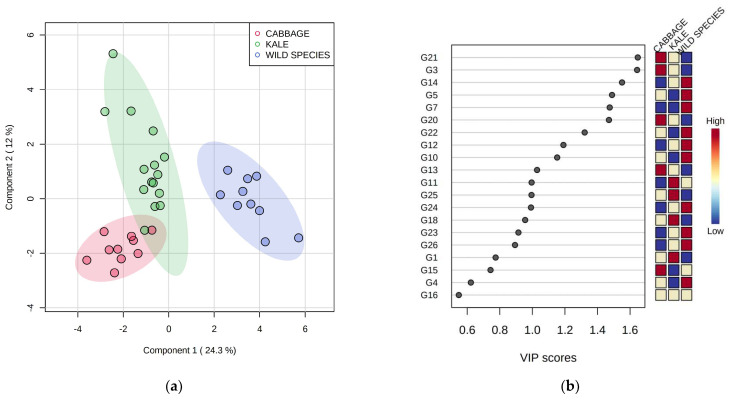
(**a**) Differentiation of Brassicaceae species in two-dimensional space by partial least squares–discriminant analysis (PLS–DA); (**b**) variable importance in projection (VIP) scores of volatile compounds (variables) generated in the glucosinolate (GSL) pathway most useful for the differentiation. Codes indicating compounds correspond to those reported in Table 1.

PLS-DA applied on GSL volatiles data resulted with a relatively good separation of the three categories: cabbage landraces, kale accessions, and wild species (Figure 4a). Two volatile compounds with the highest VIP scores, allyl nitrile (G21) and 4-methylpentanenitrile (G3), were most abundant in cabbage (Figure 4b). Among other compounds with relatively high VIP scores (>1.4), high amounts of 2-ethylthiophene (G14), 4-mercaptophenol (G5), and 2,4-pentadienenitrile (G7) were characteristic for wild species *B. incana* and *B. mollis*, while that of 3-(methylthio)butylnitrile (G20) was higher in cabbage.

### 2.4. Other Volatile Compounds

Among other compounds, cabbages contained the highest amounts of 1-butoxy-2-propanol, phenol, and 3-methylbutanal and the lowest amounts of furanoid volatiles, such as dihydroactinolide, 2-(2-propenyl)furan, and *trans*-2-(2-pentenyl)furan, as well as that of 2,6,6-trimethyl-1-cyclohexene-1-carboxaldehyde, an unidentified cyclic ketone (*m*/*z* 68, 39, 98), and 3-cyclohex-1-enyl-prop-2-enal (Table 1). The two landraces mutually differed with respect to the amounts of 1-butoxy-2-propanol and 1-octen-3-ol, both being higher in Žminjski landrace.

Several compounds differentiated particular kale accessions from the others. KAL1 contained much less 2,6,6-trimethyl-1-cyclohexene-1-carboxaldehyde and dihydroactinolide than KAL2 and KAL3, KAL2 had the highest amount of 2-(2-propenyl)furan and the unidentified cyclic ketone (*m*/*z* 68, 39, 98), and KAL3 was the most abundant in 2-phenylethanol and least abundant in an unidentified furanoid (*m*/*z* 81, 53, 96) and 4-ethyl-4-methyl-1-hexene.

An unidentified compound (*m*/*z* 113, 45, 73, 86) and tentatively identified N-isobutylidene cyclopropylamine were found in the highest amount in wild species, with a higher amount of the former in *B. incana* and the latter in *B. mollis*. The two wild species were abundant in *trans*-2-(2-pentenyl)furan, while *B. mollis* contained the highest level of benzeneacetaldehyde and the lowest level of 1-butoxy-2-propanol among all the investigated species.

Particular volatiles, such as 2,6,6-trimethyl-1-cyclohexene-1-carboxaldehyde and dihydroactinolide, were found in high amounts in the investigated wall rocket accession.

### 2.5. Differentiation Based on the Overall Volatilome

**Figure 5:**
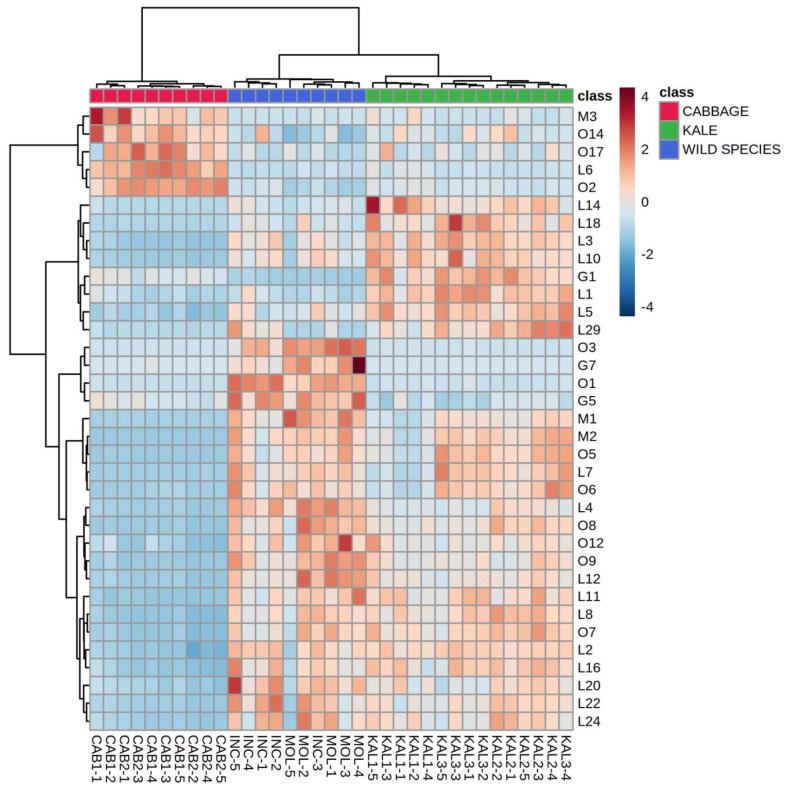
Clustering of Brassicaceae species by hierarchical cluster analysis (HCA) into three categories (cabbage, kale, and wild species *B. incana* + *B. mollis*) based on the overall composition of volatile compounds. The rows in the heatmap diagram represent compounds and the columns represent samples. Codes indicating compounds correspond to those reported in Table 1. Colors of the heatmap cells indicate low (dark blue), medium (white), and high (dark red) abundance of a particular compound.

To additionally comprehend the differences in the overall volatilome between cabbage, kale, and two wild species, HCA was applied on a reduced dataset which included all the samples grouped into three categories and all the identified volatile compounds as variables. The three categories were separated rather clearly by the amounts of the first 35 selected volatiles (Figure 5). Each category was characterized by different sets of compounds, although kale and wild species exhibited similarities in the amounts of volatile compounds clustered in the lower half of the heatmap (from M1 down), which were mostly LOX, MEP, and other volatile compounds, without a single compound from the GSL pathway. Cabbage was distinguished by higher amounts of isophorone (M3) from MEP, 2-ethyl-1-hexanol (L6) from LOX, and other miscellaneous compounds, such as phenol (O14), 3-methylbutanal (O17), and 1-butoxy-2-propanol (O2). Wild species were distinguished and grouped based on the amounts of 4-mercaptophenol (G5) and 2,4-pentadienenitrile (G7) from GSL, as well as by the amounts of the unidentified compound (*m*/*z* 113, 45, 73, 86) (O1), together with tentatively identified N-isobutylidene cyclopropylamine (O3). Kale accessions were clustered together mostly by higher amounts of several LOX generated compounds. Judging from the compounds selected from the overall volatilomes, wild species *B. incana* and *B. mollis* were more similar and were clustered much closer to kale than to cabbage. Although significant differences between landraces or accessions of the same species were found for a number of volatile compounds, HCA once again confirmed that their volatilomes retained a significant amount of important information about species’ origins.

**Figure 6:**
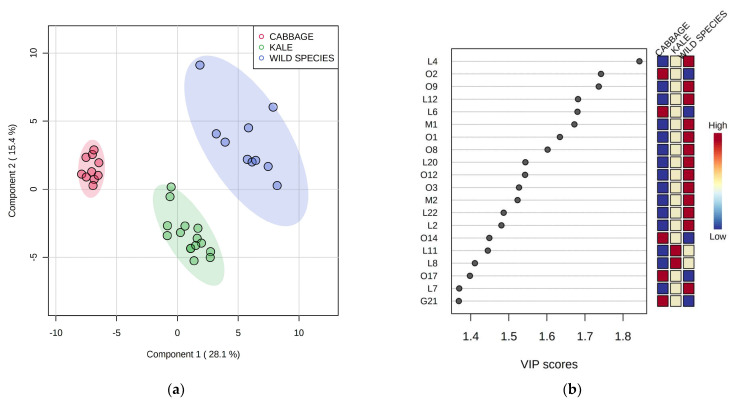
(**a**) Differentiation of Brassicaceae species in two-dimensional space by partial least squares–discriminant analysis (PLS–DA); (**b**) variable importance in projection (VIP) scores of volatile compounds (variables) from all biochemical pathways most useful for the differentiation. Codes indicating compounds correspond to those reported in Table 1.

The majority of compounds selected by PLS-DA from the overall volatilomes as those with the highest VIP scores were mostly most abundant in wild species *B. incana* and *B. mollis* and least abundant in cabbage, while kale contained medium amounts with a few exceptions (Figure 6). Again, LOX generated *trans*-2-butenal (L4), characteristic for wild species, emerged as a compound with the largest potency to discriminate the three categories. It was followed by 1-butoxy-2-propanol (O2) abundant in cabbage and *trans*-2-(2-pentenyl)furan (O9) abundant in wild species, both from the group of other volatile compounds. The compounds with relatively high VIP scores were also *trans*,*trans*-2,4-heptadienal (L12), β-ionone (M1), and two unidentified compounds with characteristic ions *m*/*z* 113, 45, 73, 86 (O1) and *m*/*z* 68, 39, 98 (O8) found in the highest level in wild species, and 2-ethyl-1-hexanol (L6) found in the highest level in cabbage.

Interestingly, LOX and other volatile compounds dominated with high VIP scores, while only a single GSL volatile, allyl nitrile (G21), was included among 20 compounds most useful for the discrimination of the three categories.

## 3. Materials and Methods

### 3.1. Brassicaceae Species, Landraces, and Accessions

Plant material was taken from six plant varieties belonging to the Brassicaceae family: cabbage (*B. oleracea* L. var. *capitata*), kale (*B. oleracea* L. var. *acephala*), Savoy cabbage (*B. oleracea* L. var. *sabauda*) cv. ‘Nebraska’, *Brassica* wild relatives (*B. incana* Ten. and *B. mollis* Vis.), and perennial wall-rocket (*D. tenuifolia* (L.) DC.). The photographs and some morphological data of the studied samples are presented in Table S1.

Cabbage samples consisted of local landraces ‘Brgujski kapuz’ (accession IPT001, CAB1) and ‘Žminjski kapuz’ (accession IPT028, CAB2) maintained as a part of the gen-bank collection of the Institute of Agriculture and Tourism in Poreč, Croatia. Both cabbage landraces included in this study are commonly grown by local farmers, with landrace ‘Brgujski’ characterized by enhanced anthocyanin coloration and landrace ‘Žminjski’ by rather slight anthocyanin coloration.

Kale samples consisted of three accessions belonging to two different morphotypes, one with blistered leaves (accessions IPT408, KAL1, and IPT418, KAL2) and one with flat leaves (accession IPT379, KAL3). All three included kale accessions are local populations previously collected from farmers and are also maintained as a part of the mentioned gen-bank collection.

Seeds of wild relatives *B. incana* (accession IPT515, INC) and *B. mollis* (accession IPT517, MOL) were collected at their natural habitats at Vis and Koločep islands, Adriatic seaside, Croatia, respectively. Seeds were germinated and plants were grown in growth chambers at 24 °C under a photoperiod 16/8 (day/night) over two months. Next, plants were transferred to a greenhouse for an adaptation period, and after two months they were transferred outside of the greenhouse.

All the plants were cultivated in the experimental field of the Institute of Agriculture and Tourism. *Diplotaxis tenuifolia* (accession IPT551, WR) was collected from a wild population growing under very similar pedoclimatic conditions near the Institute’s experimental field.

All the samples were harvested manually from 30 October to 17 November 2020. During sampling, five leaves were taken from the top portion of five fully formed, solid, and undamaged heads of the two cabbage landraces. Five samples, each consisting of two young, healthy leaves, were taken from mature plants of three kale accessions, savoy cabbage, and two wild species. Samples of *D. tenuifolia* consisted of 10 fully developed, undamaged leaves from mature plants.

### 3.2. Analysis of Volatile Compounds

Leaf samples were homogenized by a commercial blender for 30 s and 1 g of each was weighed into a glass vial of 10 mL. Four mL of deionized water was added, and the vial was sealed with a screw aluminum cap and silicone/PTFE septa. A divinylbenzene/carboxen/polydimethylsiloxane (DVB-CAR-PDMS, StableFlex, 50/30 μm, 1 cm) SPME fiber (catalog number 57328-U, Supelco, Bellafonte, PA, USA) was preconditioned above the sample for 5 min at 40 °C and then subjected to the vapors in the headspace for 15 min at 40 °C with stirring (800 rpm). Volatile compounds were desorbed in a GC/MS injector at 248 °C for 10 min (3 min splitless mode). GC/MS identification and quantification was performed using a Varian 3900 gas chromatograph (GC) connected to a Varian Saturn 2100T mass spectrometer with an ion trap analyzer (Varian Inc., Harbour City, CA, USA). The GC was equipped with a 60 m × 0.25 mm i.d. × 0.25 μm d.f. capillary column Rtx-WAX (catalog number RTK-12426, Restek, Belafonte, PA, USA). The GC column was initially heated at 40 °C, the temperature was then increased at 3.8 °C/min to 200 °C, then raised by 30 °C/min to 240 °C and kept constant for the final 15 min. The carrier gas was helium at a flow rate of 1.2 mL/min. EI mode (70 eV) was used to acquire mass spectra in the 30–350 *m*/*z* range. Volatile compounds were identified by comparison of their mass spectra with mass spectra from NIST05 library. Spectra reverse match number (RM) higher than 800 was used as a criterion, whereas in particular cases for spectra with RM < 800 the identification was performed based on the satisfactory ratio of relative intensities of a quantifier ion and the next three characteristic ions with the highest intensity. Additional confirmation of the identity of volatile compounds was achieved by comparing linear retention indices calculated relative to the retention times of C_10_ to C_28_ n-alkanes to those reported in literature obtained using equal or equivalent capillary columns. The average values of peak area divided by 1000 obtained after five replicate analyses (*n* = 5) were used in further data elaboration and reporting.

### 3.3. Statistical Data Elaboration

Data obtained by GC/MS were subjected to analysis of variance (one-way ANOVA) and the average values were compared by least significant difference (LSD) post hoc test at *p* < 0.05 level. The data were further processed by multivariate statistical analysis using hierarchical cluster analysis (HCA) and partial least squares–discriminant analysis (PLS-DA). HCA is an unsupervised multivariate statistical method in which distances between samples (cases) are calculated and samples are grouped into categories based on similar characteristics defined by the variables’ (e.g., volatile compounds’) values. PLS-DA is a supervised multivariate statistical method that minimizes the variance within and maximizes the variance between different categories (e.g., species) and gives information about the most useful variables (e.g., volatile compounds) in the form of variable importance in projection (VIP) scores. Statistica v. 13.2 software (StatSoft Inc., Tulsa, OK, USA) was used to perform ANOVA, whereas MetaboAnalyst v. 5.0 (http://www.metaboanalyst.ca, accessed on 24 October 2022) was applied for HCA and PLS-DA.

## 4. Conclusions

The HS-SPME-GC/MS method used in this study allowed for a detailed characterization of the volatilomes of several Brassicaceae species, landraces, and accessions. Each of them exhibited many unique features and volatile markers that differentiated the species among each other well, and also the landraces and accessions of the same species. On the other hand, many specific similarities between the volatilomes of different species were observed. In general, two cabbage landraces emitted the lowest amounts of volatiles generated in the LOX pathway. Wild species *B. incana* and *B. mollis* were characterized by relatively high *trans*-2-hexenal/*cis*-3-hexen-1-ol ratio compared to the other investigated vegetables. The investigated Savoy cabbage cultivar and three kale accessions exhibited particular similarities in the composition of LOX volatiles, while the LOX volatilome fraction in *B. incana* and *B. mollis* partially coincided with that of another wild species, *D. tenuifolia*. Regarding the volatiles formed in the GSL pathway, Savoy cabbage and the three wild species showed more intense emission of ITCs than cabbage landraces and kale accessions. *Diplotaxis tenuifolia* showed rather limited production of nitriles. Judging from their overall volatilomes, wild species *B. incana* and *B. mollis* turned out to be more similar to kale than to cabbage. The results of this study contribute to the general knowledge about volatile composition of vegetables from the Brassicaceae family and contribute to a better understanding of the potential of particular Brassicaceae species, landraces, and accessions in responding to stimuli from the environment. Additionally, the information obtained could be useful for better understanding the sensory quality of the investigated Brassicaceae species, which could be exploited for their better valorization. Future studies should focus on the influence of various environmental, cultivation, and post-harvest storage factors to obtain data with a higher level of applicability in practice.

## Figures and Tables

**Table 1:** Amounts of volatile compounds * found in various Brassicaceae species after headspace solid-phase microextraction followed by gas chromatography/mass spectrometry (HS-SPME-GC/MS) sorted by compound class and descending Fisher *F*-ratio.

Code	Coumpound Class and Name	LRI-Exp	LRI-Lit	*F*-Ratio	CAB1	CAB2	KAL1	KAL2	KAL3	SC	INC	MOL	WR
*Mevalonic acid (MVA) and methylerythritol phosphate (MEP) pathways volatile compounds*													
M1	β-Ionone	1940	1952	26.80	11.4 ^d^	14.2 ^d^	111.8 ^cd^	298.2 ^b^	315.4 ^b^	178.6 ^c^	347.6 ^b^	578.2 ^a^	367.2 ^b^
M2	5,6-Epoxy-β-ionone	1992	1989	15.71	2.1 ^c^	2.5 ^c^	39.4 ^b^	93.4 ^a^	99.4 ^a^	55.8 ^b^	85.1 ^a^	98.9 ^a^	93.4 ^a^
M3	Isophorone	1594	1593	8.91	169.5 ^a^	129.1 ^a^	43.8 ^b^	16.2 ^b^	9.8 ^b^	123.7 ^a^	10.9 ^b^	6.0 ^b^	10.3 ^b^
M4	Perhydrofarnesyl acetone	2133	2129	8.31	1.6 ^bc^	1.3 ^bc^	7.7 ^a^	0.9 ^bc^	1.5 ^bc^	0.7 ^c^	2.6 ^bc^	3.4 ^b^	7.2 ^a^
M5	Eucalyptol	1210	1209	1.21	7.0	5.0	1.1	5.6	3.6	0.7	0.6	4.2	1.5
M6	Lilial	2045	n.a.	0.89	3.1	2.8	3.0	2.0	2.5	2.0	2.4	2.5	2.0
*Lipoxygenase (LOX) pathway volatile compounds*													
L1	*cis*-3-Hexen-1-ol	1385	1386	43.53	42,875 ^de^	23,776 ^e^	137,317 ^b^	142,105 ^b^	206,377 ^a^	144,606 ^b^	63,475 ^cd^	25,835 ^e^	73,140 ^c^
L2	1-Penten-3-ol	1153	1155	40.19	512 ^e^	369 ^e^	1143 ^bc^	1186 ^ab^	1260 ^ab^	1240 ^ab^	1332 ^a^	988 ^cd^	885 ^d^
L3	*trans*-2-Penten-1-ol	1309	1307	29.75	23.5 ^d^	10.4 ^d^	142.6 ^ab^	144.6 ^ab^	164.5 ^a^	171.9 ^a^	122.4 ^b^	70.1 ^c^	66.0 ^c^
L4	*trans*-2-Butenal	1045	1037	29.21	2.8 ^d^	1.6 ^d^	11.8 ^c^	18.2 ^b^	10.9 ^c^	11.0 ^c^	26.2 ^a^	27.3 ^a^	19.0 ^b^
L5	3,5,5-Trimethyl-2-hexene	1482	n.a.	28.35	6.4 ^ef^	4.7 ^f^	18.9 ^bc^	19.2 ^bc^	22.4 ^b^	17.1 ^bcd^	14.1 ^cd^	11.8 ^de^	39.8 ^a^
L6	2-Ethyl-1-hexanol	1487	1486	27.56	158.0 ^a^	139.9 ^a^	38.3 ^bc^	17.5 ^c^	19.6 ^bc^	23.8 ^bc^	11.7 ^c^	16.5 ^c^	49.6 ^b^
L7	6-Methyl-5-hepten-2-ol	1620	n.a.	23.37	0.5 ^e^	0.5 ^e^	6.1 ^de^	21.9 ^c^	29.8 ^ab^	13.2 ^d^	23.3 ^bc^	21.1 ^c^	33.0 ^a^
L8	*trans*,*cis*-2,4-Heptadienal	1462	1460	22.00	102.1 ^c^	45.9 ^c^	448.4 ^b^	615.1 ^a^	490.6 ^b^	534.8 ^ab^	468.0 ^b^	481.4 ^b^	531.4 ^ab^
L9	3,5,5-Trimethyl-3-cyclohexanone	1411	1410	19.95	8.5 ^b^	4.2 ^bcd^	6.9 ^bc^	1.1 ^cd^	1.0 ^cd^	29.7 ^a^	0.5 ^d^	0.3 ^d^	0.2 ^d^
L10	*trans*-3-Penten-2-one	1120	1121	19.10	1.4 ^e^	0.8 ^e^	13.9 ^b^	11.9 ^b^	13.2 ^b^	21.1 ^a^	9.6 ^bc^	6.5 ^cd^	3.7 ^de^
L11	3,4-Nonadiene	2054	n.a.	18.92	3.2 ^c^	2.9 ^c^	31.2 ^b^	32.3 ^b^	35.9 ^ab^	43.7 ^a^	29.9 ^b^	37.4 ^ab^	7.7 ^c^
L12	*trans*,*trans*-2,4-Heptadienal	1491	1487	18.52	204 ^d^	101 ^d^	1019 ^bc^	1100 ^bc^	897 ^bc^	1186 ^b^	1151 ^b^	1828 ^a^	795 ^c^
L13	2-Methyl-2-pentenal	<1000	982	17.94	3.8 ^d^	0.6 ^d^	18.6 ^d^	74.2 ^b^	51.8 ^bc^	16.2 ^d^	48.6 ^c^	12.1 ^d^	110.3 ^a^
L14	3-Ethyl-1,5-octadiene	1037	1027	15.27	2.4 ^e^	0.0 ^e^	79.6 ^a^	52.9 ^b^	29.9 ^cd^	49.5 ^bc^	19.6 ^de^	10.8 ^de^	3.5 ^e^
L15	*trans*-3-Hexen-1-ol	1361	1358	15.06	1910 ^b^	1893 ^b^	16,505 ^b^	2336 ^b^	9488 ^b^	70,947 ^a^	716 ^b^	2624 ^b^	2347 ^b^
L16	*cis*-4-Heptenal	1240	1240	13.83	2.6 ^c^	1.4 ^c^	8.6 ^ab^	10.4 ^a^	9.0 ^ab^	10.5 ^a^	10.1 ^a^	6.9 ^b^	8.0 ^ab^
L17	Alkene, n.i. *m*/*z* 55, 70, 41	1246	n.a.	13.55	28.0 ^c^	33.7 ^bc^	39.0 ^b^	34.4 ^bc^	44.2 ^ab^	53.5 ^a^	27.6 ^c^	16.7 ^d^	8.4 ^d^
L18	3-Pentanone	1002	1002	13.10	29.5 ^c^	28.8 ^c^	434.2 ^b^	411.8 ^b^	739.6 ^a^	472.8 ^b^	198.3 ^c^	196.7 ^c^	432.4 ^b^
L19	Hexanoic acid	1846	1848	12.85	14.0 ^d^	23.3 ^d^	44.6 ^bc^	83.2 ^a^	57.0 ^b^	56.6 ^b^	47.2 ^b^	28.7 ^cd^	20.7 ^d^
L20	*trans*-2-Hexenal	1218	1219	12.08	38,717 ^ef^	30,052 ^f^	72,222 ^bcd^	85,546 ^b^	56,715 ^de^	63,388 ^cd^	110,718 ^a^	81,749 ^bc^	89,090 ^b^
L21	1-Hexanol	1351	1353	11.76	6266 ^cd^	10,344 ^b^	9036 ^bc^	4689 ^de^	15,905 ^a^	10,719 ^b^	1788 ^ef^	375 ^f^	5176 ^cde^
L22	*trans*-2-Pentenal	1123	1121	11.67	67.5 ^d^	36.6 ^d^	283.0 ^c^	414.8 ^ab^	309.3 ^bc^	369.6 ^bc^	530.1 ^a^	317.9 ^bc^	264.5 ^c^
L23	*cis*-3-Hexenal	1132	1134	10.70	945 ^a^	299 ^bcde^	282 ^cde^	285 ^cde^	147 ^e^	166 ^de^	498 ^b^	359 ^bcd^	425 ^bc^
L24	1-Penten-3-one	1031	1031	10.57	331 ^d^	185 ^d^	1654 ^ab^	2194 ^a^	1274 ^bc^	2367 ^a^	2075 ^a^	1765 ^ab^	719 ^cd^
L25	Nonanoic acid	2164	2168	9.30	11.0 ^b^	6.7 ^bc^	3.9 ^bc^	27.8 ^a^	3.6 ^c^	2.9 ^c^	2.8 ^c^	4.7 ^bc^	5.4 ^bc^
L26	*trans*-2-Hexen-1-ol	1403	1406	8.86	352.6 ^cd^	286.5 ^cd^	2218.1 ^b^	1096.6 ^bcd^	3917.1 ^a^	1343.3 ^bc^	706.7 ^cd^	97.4 ^d^	874.0 ^cd^
L27	5-Methyl-3-heptene	1114	n.a.	8.33	0.7 ^c^	0.3 ^c^	1.5 ^bc^	2.4 ^b^	1.1 ^bc^	2.3 ^b^	0.5 ^c^	0.5 ^c^	4.8 ^a^
L28	*cis*-2-Hexenal	1198	1196	7.16	315 ^cd^	252 ^d^	376 ^cd^	447 ^bc^	257 ^d^	296 ^d^	651 ^a^	374 ^cd^	529 ^ab^
L29	*trans*-3-Hexenoic acid	1947	1948	7.13	0.5 ^d^	0.4 ^d^	3.4 ^cd^	8.5 ^ab^	6.4 ^bc^	2.9 ^cd^	4.2 ^cd^	0.6 ^d^	10.6 ^a^
L30	*trans*-6-Nonenal	1535	1535	6.75	4.0 ^bc^	3.8 ^bc^	6.0 ^a^	4.8 ^abc^	3.7 ^bcd^	5.1 ^ab^	1.7 ^e^	3.5 ^cd^	2.3 ^de^
L31	Octanal	1288	1289	6.54	1.3 ^bc^	2.4 ^ab^	3.6 ^a^	0.8 ^c^	3.5 ^a^	0.9 ^c^	0.8 ^c^	0.8 ^c^	3.4 ^a^
L32	*trans*-2-Octenal	1429	1427	6.35	26.2 ^bcd^	19.7 ^cd^	42.5 ^a^	27.9 ^bc^	23.1 ^cd^	36.1 ^ab^	15.8 ^de^	26.8 ^bcd^	7.4 ^e^
L33	*trans*,*trans*-2,4-Hexadienal	1395	1391	5.19	220 ^bc^	195 ^bcd^	241 ^bc^	176 ^cd^	183 ^cd^	126 ^d^	318 ^a^	180 ^cd^	254 ^ab^
L34	Heptanal	1182	1179	5.08	5.6 ^d^	6.2 ^d^	9.7 ^ab^	7.6 ^bcd^	6.8 ^cd^	12.2 ^a^	7.4 ^bcd^	5.7 ^d^	9.2 ^bc^
L35	*cis*-2-Heptenal	1323	1322	4.46	27.4 ^cd^	37.1 ^bcd^	53.5 ^ab^	54.0 ^ab^	58.3 ^a^	44.6 ^abc^	42.2 ^abc^	52.4 ^ab^	19.3 ^d^
L36	*cis*-3-Hexenyl isovalerate	1475	1480	4.30	0.0 ^b^	0.0 ^b^	2.4 ^b^	1.1 ^b^	7.5 ^a^	0.9 ^b^	2.3 ^b^	0.0 ^b^	0.9 ^b^
L37	*trans*,*trans*-2,4-Nonadienal	1714	1712	3.77	8.3 ^b^	10.5 ^b^	15.9 ^b^	13.4 ^b^	11.2 ^b^	15.8 ^b^	8.1 ^b^	15.6 ^b^	106.9 ^a^
L38	Hexanal	1080	1079	3.57	12,152 ^bc^	20,079 ^a^	13,801 ^abc^	15,286 ^abc^	13,547 ^bc^	15,379 ^ab^	16,562 ^ab^	8898 ^cd^	5543 ^d^
L39	2,2-Dimethyl-3-hexene	1515	n.a.	3.40	4.8 ^b^	4.2 ^b^	8.0 ^b^	7.7 ^b^	4.6 ^b^	5.6 ^b^	10.4 ^b^	5.2 ^b^	18.8 ^a^
L40	*trans*-3-Hexenal	1138	1138	2.82	1383 ^abc^	1377 ^abc^	1057 ^bc^	825 ^bc^	433 ^c^	560 ^c^	2177 ^a^	684 ^bc^	1534 ^ab^
L41	*cis*-2-Penten-1-ol	1316	1318	2.56	1498 ^b^	1984 ^b^	13,820 ^a^	10,297 ^ab^	13,541 ^a^	7816 ^ab^	14,686 ^a^	5781 ^ab^	4044 ^b^
L42	*cis*-2-Pentenal	1100	1105	1.42	7.5	5.7	24.7	31.8	81.4	27.4	57.6	22.3	26.2
L43	*trans*,*cis*-2,4-Hexadienal	1401	1395	1.35	475	412	438	498	653	362	790	498	554
	Total LOX compounds				108,713 ^de^	92,044 ^e^	272,937 ^b^	270,227 ^b^	326,842 ^a^	322,559 ^a^	219,390 ^c^	133,442 ^d^	187,727 ^c^
*Glucosinolate pathway (GSL) volatile compounds*													
G1	Methyl thiocyanate	1269	1274	29.73	129.0 ^b^	104.6 ^bc^	252.0 ^a^	292.1 ^a^	308.8 ^a^	279.9 ^a^	14.9 ^d^	0.0 ^d^	44.4 ^cd^
G2	4-Methylthiazole	1282	1287	21.42	8.5 ^bc^	6.1 ^bc^	34.2 ^b^	8.0 ^bc^	34.2 ^b^	160.7 ^a^	0.0 ^c^	3.7 ^bc^	0.1 ^c^
G3	4-Methylpentanenitrile	1243	1253	21.06	416.1 ^a^	149.4 ^b^	200.9 ^b^	40.1 ^c^	41.7 ^c^	24.2 ^c^	8.0 ^c^	3.2 ^c^	8.1 ^c^
G4	4-Methylpentyl isothiocyanate	1544	1533	18.96	2.2 ^b^	1.6 ^b^	3.5 ^b^	0.6 ^b^	1.6 ^b^	2.7 ^b^	1.9 ^b^	12.6 ^b^	77.7 ^a^
G5	4-Mercaptophenol	1897	n.a.	17.32	1.8 ^b^	1.6 ^bc^	1.2 ^bcd^	1.5 ^bc^	0.8 ^d^	0.8 ^cd^	3.8 ^a^	3.6 ^a^	1.9 ^b^
G6	Allyl isothiocyanate	1359	1353	14.83	615.9 ^bc^	403.3 ^bc^	3962.4 ^b^	754.1 ^bc^	3228.0 ^bc^	15,951.0 ^a^	20.8 ^c^	597.0 ^bc^	5.3 ^c^
G7	2,4-Pentadienenitrile	1262	n.a.	14.34	1.9 ^c^	0.0 ^c^	0.0 ^c^	0.0 ^c^	0.0 ^c^	4.9 ^c^	27.7 ^b^	90.3 ^a^	0.5 ^c^
G8	Isothiocyanate derivative n.i. *m*/*z* 99, 41, 39, 72	1449	n.a.	13.25	4.4 ^b^	5.3 ^b^	23.7 ^b^	5.9 ^b^	18.7 ^b^	102.8 ^a^	13.5 ^b^	2.9 ^b^	2.3 ^b^
G9	1-Allyl-1-cyclohexanol	1571	n.a.	9.64	7.9 ^b^	5.0 ^bc^	4.0 ^bc^	16.6 ^a^	13.9 ^a^	3.8 ^bc^	5.7 ^bc^	1.9 ^c^	12.8 ^a^
G10	Benzenepropanenitrile	2034	2041	8.68	210.1 ^b^	637.4 ^b^	476.6 ^b^	93.1 ^b^	625.2 ^b^	824.2 ^b^	704.1 ^b^	2864.8 ^a^	16.8 ^b^
G11	Unsatur. aliph. thiol, n.i. *m*/*z* 41, 68, 69, 39	1555	n.a.	8.35	3.3 ^e^	5.1 ^e^	17.3 ^de^	32.1 ^cd^	56.4 ^ab^	70.3 ^a^	18.5 ^de^	45.3 ^bc^	4.6 ^e^
G12	3-Butenyl isothiocyanate	1455	1453	8.25	6.5 ^b^	1.5 ^b^	79.8 ^b^	7.2 ^b^	135.2 ^b^	286.4 ^b^	9516.0 ^b^	74,056.8 ^a^	163.8 ^b^
G13	Carbon disulfide	<1000	735	8.13	65.5 ^bc^	46.6 ^cd^	103.4 ^ab^	16.0 ^de^	39.3 ^cde^	47.1 ^cd^	5.9 ^e^	13.0 ^de^	115.2 ^a^
G14	2-Ethylthiophene	1171	1173	8.03	52.2 ^c^	38.9 ^c^	217.4 ^b^	177.0 ^bc^	155.9 ^bc^	65.2 ^c^	502.3 ^a^	216.7 ^b^	37.2 ^c^
G15	Hexanenitrile	1297	1303	7.91	32.6 ^a^	25.6 ^ab^	16.8 ^bcd^	7.2 ^def^	4.0 ^ef^	6.9 ^def^	13.5 ^cde^	23.8 ^abc^	1.7 ^f^
G16	Erucin	2137	2132	7.80	0.0 ^b^	0.0 ^b^	0.0 ^b^	0.0 ^b^	0.0 ^b^	0.0 ^b^	0.0 ^b^	47.3 ^b^	12,891.0 ^a^
G17	Cycl. sulfur compound, n.i. *m*/*z* 88, 116, 117, 60	1846	n.a.	7.50	0.2 ^b^	0.5 ^b^	0.0 ^b^	0.6 ^b^	0.9 ^b^	0.2 ^b^	0.0 ^b^	0.0 ^b^	196.9 ^a^
G18	2-Methylbutyl isothiocyanate	1423	1412	7.00	27.2 ^bcd^	28.0 ^bcd^	48.9 ^b^	14.9 ^cd^	31.5 ^bc^	79.2 ^a^	0.0 ^d^	0.1 ^d^	0.0 ^d^
G19	Iberverin	1982	1979	6.62	14.4 ^b^	2.9 ^b^	23.8 ^b^	2.7 ^b^	11.7 ^b^	21.8 ^b^	0.4 ^b^	5.0 ^b^	300.0 ^a^
G20	3-(Methylthio)butylnitrile	1799	1806	5.36	206.7 ^a^	102.6 ^bc^	118.1 ^b^	91.7 ^bc^	60.6 ^bcd^	21.5 ^cd^	0.4 ^d^	3.4 ^d^	0.7 ^d^
G21	Allyl nitrile	1173	1186	5.31	934.2 ^ab^	1096.5 ^a^	673.4 ^ab^	488.2 ^bc^	501.7 ^bc^	808.0 ^ab^	0.5 ^c^	3.8 ^c^	0.6 ^c^
G22	4-(Methylthio)butylnitrile	1937	1931	5.08	1.1 ^bc^	0.4 ^c^	0.8 ^c^	0.1 ^c^	0.4 ^c^	0.3 ^c^	16.0 ^a^	9.5 ^ab^	14.3 ^a^
G23	Phenethyl isothiocyanate	2222	2216	4.29	0.7 ^b^	3.4 ^b^	28.6 ^b^	1.5 ^b^	25.6 ^b^	395.0 ^a^	17.3 ^b^	352.1 ^a^	23.1 ^b^
G24	Benzyl isothiocyanate	2098	2107	3.88	15.1 ^b^	3.9 ^b^	60.4 ^b^	3.7 ^b^	16.4 ^b^	17.0 ^b^	64.5 ^b^	46.5 ^b^	377.8 ^a^
G25	Isothiocyanate derivative, n.i. *m*/*z* 99, 71, 72, 59	1837	n.a.	3.20	1010.9 ^abc^	1298.2 ^a^	1748.2 ^a^	1213.7 ^ab^	1184.1 ^ab^	1456.8 ^a^	19.1 ^c^	152.8 ^bc^	0.0 ^c^
G26	Benzyl nitrile	1923	1927	2.76	94.5 ^b^	11.8 ^b^	533.1 ^a^	54.0 ^b^	92.4 ^b^	14.7 ^b^	570.7 ^a^	268.2 ^ab^	3.2 ^b^
G27	Dimethyl disulfide	1073	1075	2.51	89.4 ^abc^	15.6 ^c^	237.1 ^a^	45.6 ^bc^	130.3 ^abc^	197.9 ^ab^	22.2 ^c^	6.6 ^c^	13.1 ^c^
G28	Isothiocyanatocyclopropane	1229	1223	0.84	1839.5	7.5	54.4	10.3	52.8	254.9	4268.7	4.7	1135.1
	Total isothiocyanates (ITCs)				3537 ^d^	1756 ^d^	6034 ^cd^	2015 ^d^	4706 ^d^	18,568 ^b^	13,922 ^bc^	75,278 ^a^	14,976 ^bc^
	Total nitriles				1897.1 ^bc^	2023.7 ^b^	2019.7 ^b^	774.5 ^c^	1326.0 ^bc^	1704.7 ^bc^	1341.0 ^bc^	3267.0 ^a^	46.0 ^d^
	Total ITCs/total nitriles				1.92 ^c^	0.88 ^c^	2.57 ^c^	2.31 ^c^	3.62 ^c^	11.43 ^c^	10.06 ^c^	31.09 ^b^	315.34 ^a^
*Other compounds*													
O1	n.i. *m*/*z* 113, 45, 73, 86	1938		105.30	17.2 ^c^	7.3 ^c^	81.6 ^c^	26.1 ^c^	95.1 ^c^	50.0 ^c^	1848.5 ^a^	1267.1 ^b^	1.9 ^c^
O2	1-Butoxy-2-propanol	1341	1364	77.16	28.0 ^b^	35.4 ^a^	11.9 ^c^	9.1 ^cd^	9.5 ^cd^	9.4 ^cd^	9.1 ^cd^	2.3 ^e^	6.9 ^d^
O3	N-isobutylidene cyclopropylamine	1070	na	55.28	4.1 ^c^	2.9 ^c^	10.1 ^c^	1.3 ^c^	7.8 ^c^	4.0 ^c^	259.2 ^b^	471.4 ^a^	1.4 ^c^
O4	2-Phenylethanol	1907	1910	22.00	9.4 ^bc^	12.8 ^bc^	56.3 ^bc^	21.5 ^bc^	194.6 ^a^	240.5 ^a^	68.8 ^b^	232.9 ^a^	6.5 ^c^
O5	1-Cyclohexene-1-carboxaldehyde	1627	1631	21.83	1.4 ^e^	1.5 ^e^	14.2 ^d^	33.4 ^ab^	40.2 ^a^	21.6 ^cd^	24.5 ^bc^	33.2 ^ab^	36.8 ^a^
O6	Dihydroactinolide	2354	2332	18.43	0.6 ^d^	0.1 ^d^	3.0 ^cd^	11.3 ^ab^	12.5 ^ab^	4.9 ^c^	10.8 ^ab^	9.3 ^b^	13.2 ^a^
O7	2-(2-Propenyl)furan	1615	n.a.	18.23	2.9 ^c^	1.5 ^c^	12.2 ^b^	15.5 ^a^	12.0 ^b^	14.2 ^ab^	11.0 ^b^	13.0 ^ab^	11.3 ^b^
O8	Cyclic ketone, n.i. *m*/*z* 68, 39, 98	1705	n.a.	13.59	9.3 ^e^	5.3 ^e^	42.4 ^d^	66.4 ^ab^	45.7 ^cd^	54.1 ^abcd^	60.8 ^abc^	69.9 ^a^	50.3 ^bcd^
O9	*trans*-2-(2-Pentenyl)furan	1302	1282	13.18	0.8 ^d^	0.4 ^d^	4.2 ^bc^	4.4 ^bc^	4.4 ^bc^	4.7 ^bc^	6.0 ^ab^	7.4 ^a^	3.0 ^c^
O10	3-Cyclohex-1-enyl-prop-2-enal	1890	n.a.	10.45	3.5 ^ef^	2.2 ^f^	14.4 ^bc^	8.6 ^cde^	9.5 ^bcd^	22.9 ^a^	11.7 ^bc^	15.3 ^b^	4.6 ^def^
O11	Benzeneacetaldehyde	1646	1648	9.85	10.4 ^c^	29.4 ^c^	33.1 ^c^	19.7 ^c^	108.6 ^bc^	260.0 ^b^	163.6 ^bc^	623.3 ^a^	8.9 ^c^
O12	Benzaldehyde	1522	1521	9.23	49.9 ^cd^	27.8 ^d^	92.4 ^bc^	92.0 ^bc^	76.5 ^cd^	111.4 ^ab^	101.9 ^abc^	122.1 ^a^	77.0 ^cd^
O13	Furanoid, n.i. *m*/*z* 81, 53, 96	<1000	na	6.54	183 ^cde^	152 ^e^	257 ^bc^	247 ^bc^	165 ^de^	169 ^de^	359 ^a^	237 ^bcd^	274 ^b^
O14	Phenol	1995	1995	5.29	15.3 ^a^	13.5 ^ab^	10.7 ^c^	11.2 ^bc^	10.1 ^cd^	10.8 ^c^	10.2 ^cd^	7.8 ^d^	11.6 ^bc^
O15	4-Ethyl-4-methyl-1-hexene	1955	na	4.92	170 ^cde^	139 ^e^	214 ^bcd^	215 ^bcd^	128 ^e^	155 ^de^	287 ^a^	237 ^abc^	240 ^ab^
O16	1-Octen-3-ol	1447	1447	4.29	35.0 ^bcd^	49.5 ^a^	48.1 ^a^	38.3 ^abc^	44.1 ^ab^	46.6 ^ab^	24.2 ^d^	31.0 ^cd^	31.3 ^cd^
O17	3-Methylbutanal	<1000	924	3.48	1.2 ^a^	1.2 ^a^	0.4 ^b^	0.3 ^b^	0.3 ^b^	0.7 ^ab^	0.2 ^b^	0.1 ^b^	0.3 ^b^
O18	2-Phenoxyethanol	2144	2144	2.11	6.8	6.6	115.8	7.8	8.7	6.8	27.7	30.4	24.0
O19	Isopropyl myristate	2038	2040	1.76	18.1	17.5	11.8	10.5	9.8	8.4	9.3	234.1	15.8

* Amounts of volatile compounds are reported as peak area/1000 obtained as mean values of five replicate analyses (*n* = 5). Volatile compounds were identified by comparison of mass spectra to those from NIST05 mass spectra electronic library and by comparison of linear retention indexes calculated relative to *n*-alkanes (LRI-exp) to those from literature (LRI-lit); statistically significant differences between mean values at *p* < 0.05, obtained by one-way ANOVA and least significant difference (LSD) test, are marked by different superscript lowercase letters in a row. Abbreviations: CAB1—cabbage, *B. oleracea* L. var. *capitata*, landrace Brgujski, accession IPT001; CAB2—cabbage, *B. oleracea* L. var. *capitata*, landrace Žminjski, accession IPT028; KAL1—kale, *B. oleracea* var. *acephala*, accession IPT408; KAL2—kale, *B. oleracea* var. *acephala*, accession IPT418; KAL3—kale, *B. oleracea* var. *acephala*, accession IPT379; SC—Savoy cabbage, *B. oleracea* var. *sabauda*, variety Nebraska; INC—*Brassica* wild relative, *B. incana* Ten., accession IPT515; MOL—*Brassica* wild relative, *B. mollis* Vis., accession IPT517; WR—wall rocket/rocket salad, *D. tenuifolia* L., accession IPT551; n.i.—not identified.

## Data Availability

All data generated or analyzed during this study are included in this published article.

## Supplementary Materials

The following supporting information can be downloaded at: https://www.mdpi.com/article/10.3390/plants12010177/s1, Table S1: Morphological parameters of different Brassicaceae species.
