# Supplementary material for: Comprehensive Volatilome Signature of Various Brassicaceae Species
Source: Plants (Basel). 2023 Jan 1;12(1):177. doi: 10.3390/plants12010177 (PMC9824104; doi:10.3390/plants12010177)
Supplement: Supplementary file 1 [file plants-12-00177-s001.zip › plants-2115819-supplementary.pdf]

Table S1. Morphological parameters of different Brassicaceae species.

Species	Common name	Abbreviation	Accession	Picture	Morphological characteristics
Brassica oleacera L. var. *capitata*, landrace Brgujski	Cabbage	CAB1	IPT001	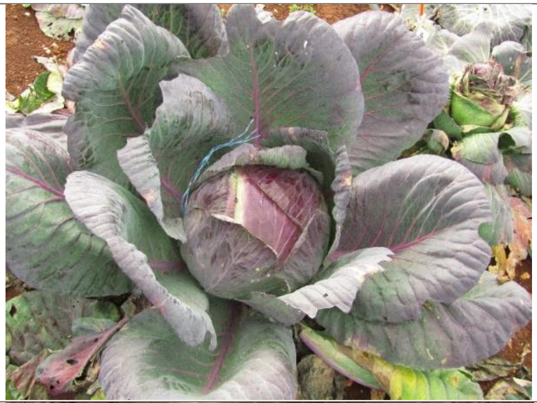	Head formation, enhanced anthocyanin coloration
Brassica oleacera L. var. *capitata*, landrace Žminjski	Cabbage	CAB2	IPT028	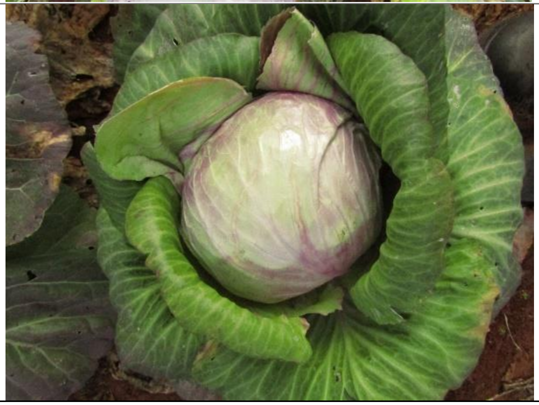	Head formation, slight anthocyanin coloration

Brassica oleracea var. *acephala*	Kale	KAL1	IPT408	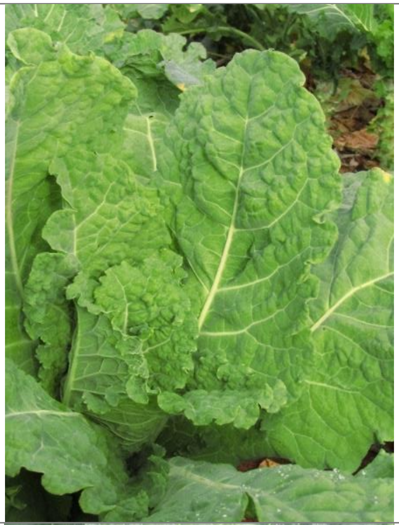	Semihead formation, blistered leaves
Brassica oleracea var. *acephala*	Kale	KAL2	IPT418	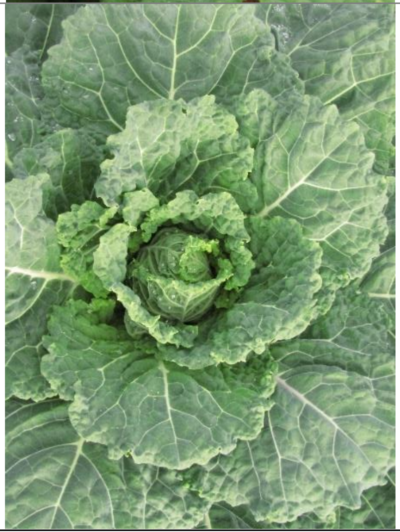	Semihead formation, blistered leaves

Brassica oleracea var. *acephala*	Kale	KAL3	IPT379	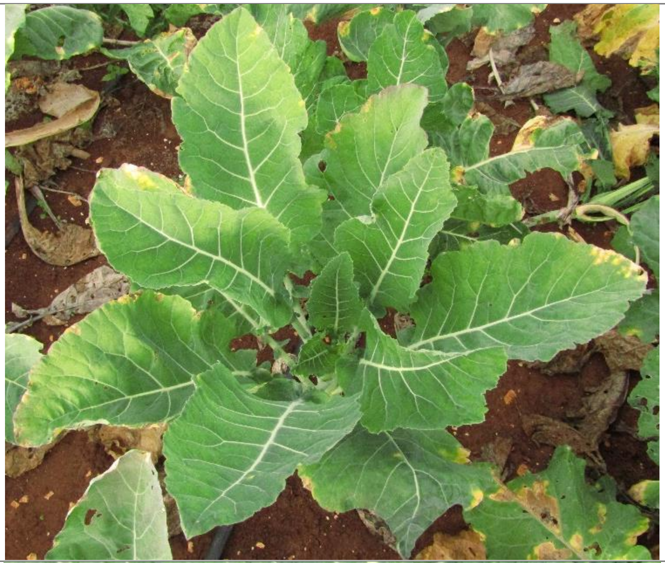	No head formation, flat leaves
Brassica oleracea var. *sabauda*, variety Nebraska	Savoy cabbage	SC	Commercial variety	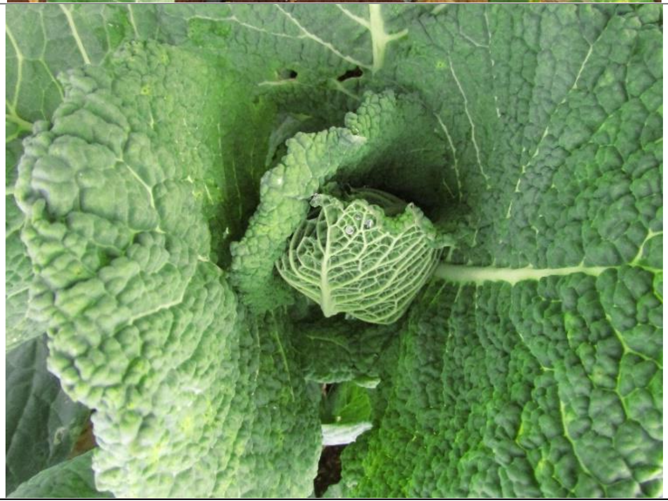	Head formation, blistered leaves

Brassica incana Ten.	n.a. (wild species)	INC	IPT515	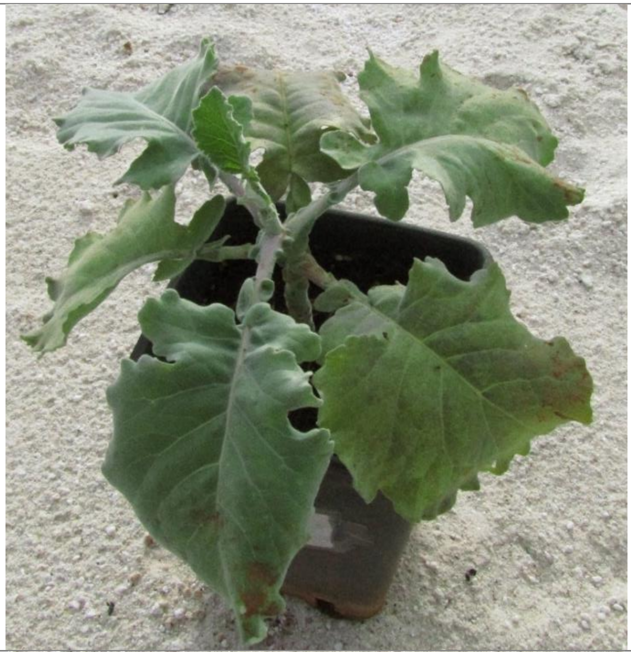	No head formation, hairy leaves
Brassica mollis Vis.	n.a. (wild species)	MOL	IPT517	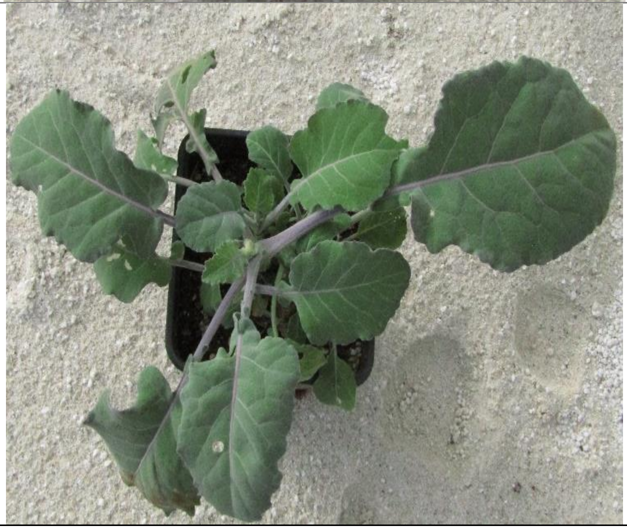	No head formation, hairy leaves

Diplotaxis tenuifolia L., wild population	Wall rocket	WR	IPT551	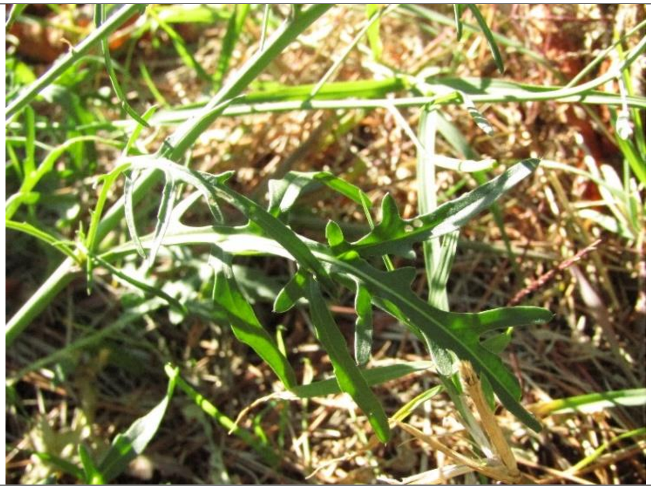	No head formation, small and deeply divided leaves
